# A Metatitanic Acid Particulate Xerogel: Green Synthesis, Structure Determination, and Detailed Characterization

**DOI:** 10.1021/acs.inorgchem.4c00369

**Published:** 2024-06-29

**Authors:** Monika Motlochová, Xenia Vislocká, Sven Lidin, Mária Čaplovičová, Roman Maršálek, Jan Šubrt

**Affiliations:** † Institute of Inorganic Chemistry of the Czech Academy of Sciences, 250 68 Řež, Czech Republic; ‡ Centre for Analysis and Synthesis, Lunds Universitet, Naturvetarvägen 14, Lund 222-61, Sweden; § Slovak University of Technology in Bratislava, Faculty of Material Science and Technology, Centre for Nanodiagnostics of Materials, Vazovova 5, Bratislava 81243, Slovakia; ∥ Department of Chemistry, Faculty of Science, University of Ostrava, CZ-701 03 Ostrava, Czech Republic

## Abstract

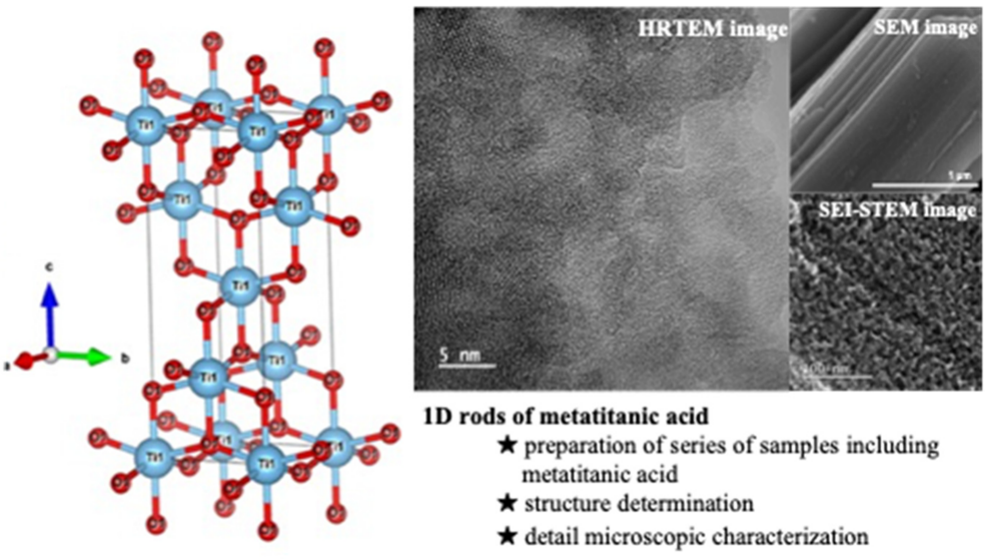

The manuscript focuses on an original method of preparation of metatitanic acid when only environmentally safe base substances are used in the synthesis process. The synthesis is based on the reaction of solid titanyl sulfate in an aqueous solution of sodium hydroxide. This method allows for (i) a full preservation of the morphology of the starting titanyl sulfate and (ii) a preparation of metatitanic acid substances with specific parameters. This can be achieved via a precise control of the alkali metal/titanyl sulfate ratio resulting in substances with varying contents of alkali metals or even sulfate anions. The prepared metatitanic acid then also contains very small weakly crystalline particles (2–3 nm) and forms pseudomorphic aggregates whose shape and dimensions correspond to those of the starting titanyl sulfate. These aggregates exhibit regular nanoporosity with a high surface area of up to 500 m^2^·g^–1^, have no tendency to form colloids, and are mechanically highly resistant even by high-energy ultrasound. The characterization of the resulting products is done via their chemical composition and methods of structural analysis, as well as by electron microscopy and local analysis. The mechanism of product formation is discussed based on the structure of the precursor, including the so far unknown structure of metatitanic acid.

## Abstract 2

The manuscript focuses on the original preparation of metatitanic acid in a nontoxic way. The method used, based on crystalline titanyl sulfate as starting material, allows (i) complete preservation of the morphology of the titanyl sulfate in the products and (ii) preparation of products with specific chemical compositions. Characterization of the products was carried out and the mechanism of product formation is discussed, including a proposed hitherto unknown structure of metatitanic acid.

## 1 Introduction

Inorganic ion exchangers have established themselves firmly among ion-exchange materials. The rapid progress in nuclear energy, hydrometallurgy of rare elements, preparation of high-purity materials, water purification, etc., has pushed for the development and synthesis of new, highly selective ion-exchanging materials resistant to chemicals, radiation, or temperature changes and with more convenient properties than commercially available organic or natural inorganic (soils, clay minerals, etc.) ion-exchangers. A large number of synthetic inorganic substances, which exhibit ion-exchanging properties, have been described. Metatitanic acid and metatitanates occupy an important place among those materials.^1−5^

This has been known for a long time, yet the use of hydrated titanium dioxide materials under real-life conditions is still rather limited. The hydrated polyvalent metal oxides, including hydrated titanium dioxide, meet all of the above-described criteria (highly selective ion-exchange materials, etc.) for an efficient compound. However, these hydroxide sorbates also have their own disadvantages, mainly limitations regarding the production of higher volumes (with stable and homogeneous properties over time) and unsatisfactory sorption kinetics of trace element extraction during the operation of the ion-exchange material. These drawbacks are driven by the gel structure of the precipitation products. Moreover, synthesized hydroxyl sorbents and active formulations are rather rare and expensive.^6^

To date, a number of papers have been published showing the significant potential of hydrated titanium dioxide and its use as a sorbent with inorganic ion-exchange properties.^7−20^ It has been shown that metatitanic acid is an emerging lithium sieve that can potentially replace the well-known manganese-based materials.^9,10^ In this context, it is worth noting that the high sorption capacity of metatitanic acid was also used to design the electrodes for Li^21^ and also for Na and K ion batteries.^22^

Hydrous titanium oxide with ion-exchange properties is prepared by the mixing of titanyl oxalate or TiCI_4_ solutions with sodium hydroxide. White amorphous products are obtained which lose free or interstitial water in temperatures up to 200 °C and chemically bound water at higher temperatures (200–500 °C).^1^ A number of other synthetic processes for the preparation of metatitanic acid have been described, e.g., from Li-titanate^5^ and various sol–gel processes from organic precursors, e.g., titanium iso-propoxide^23^ or via hydrolysis using titanium sulfate and distilled water as starting materials.^24^ Metatitanic acid is also a very important intermediate in the production of titanium dioxide by sulfate technology, i.e., hydrolysis of products of ilmenite decomposition with sulfuric acid. Titanium dioxide is then produced by controlled calcination of the metatitanic acid thus prepared.^25^ This intermediate also has significant sorption properties.^6,8,26^

Recently, our team developed a preparation method for the synthesis of metatitanic acid microrods in aqueous media starting with solid hydrated titanyl sulfate crystals with defined morphology. The method is based on the extraction of sulfate ions from the crystals and their replacement with hydroxyl groups in basic aqueous solution. The particle size and morphology of the starting hydrated titanyl sulfate were closely preserved in the pseudomorphs of amorphous metatitanic acid including such details like the layered structure of the original hydrated titanyl sulfate crystals. The rod-like particles of metatitanic acid possess excellent ion-exchange properties as well as efficient sorption properties toward radionuclides and many heavy metal ions.^27−32^

The main issue preventing a better understanding of the properties of metatitanic acid as a sorbent is the lack of knowledge about its structure as well as the structure of metatitanates of various metals. The aim of this work is to describe the chemistry of the reactions involved in the synthesis of the material as well as the short-range ordering in the products.

It is clear that despite a number of promising properties, inorganic ion exchangers have not yet seen widespread commercial use. The main cited reason is that various synthetic processes produce materials in the form of fine powders or gels unsuitable for column work, and no concerted effort has yet been made to develop suitable binders or to prepare materials in the form of beads.^20^ The material discussed in this work, based on hydrated alkali metals or ammonium metatitanates, aims to overcome these shortcomings. The material can be prepared in the form of solid aggregates whose size and shape depend on the morphology of the starting hydrated titanyl sulfate used for the synthesis. Such a material could be suitable for use as an ion exchanger. Among the goals of this paper are to explore the variations in the molar ratio of alkali metal to titanyl sulfate and their effect on the composition of the final product, to prepare a material containing no alkali metal at all (corresponding to metatitanic acid composition), to describe its structure and properties, and to compare it with substances described as metatitanic acid in the literature and in technical practice.

## 2 Materials and Methods

### 2.1 Synthesis

The material was synthesized as a modification of the existing procedures,^27,33^ i.e., a total of 100 mL of cooled distilled water was mixed with 50 g of ice (made of distilled water) and 3.50, 4.20, 4.35, 4.50, and 7.00 mL of sodium hydroxide (Penta, Czech Republic), respectively (see Table 1). After the addition of 4.80 g of titanyl sulfate dihydrate (TiOSO_4_·2H_2_O, min. 29% Ti as TiO_2_ basis, technical grade purity, provided by Sigma-Aldrich, equivalent of 24.5 mmol), the suspension had a temperature of 0 °C. While the mixture was magnetically stirred for 2 h, the temperature rose to room temperature (RT). Then the suspension was decanted twice, solid residue was filtered off and washed with 400 mL of distilled water, and dried in a Petri dish at RT.

**Table 1:** Summary of Surface Area Evaluation Showing Isotherm Classification, Micropore Evaluation by the t-Plot Method, and Surface Area Evaluation by the BET Method

sample	micropore volume [cm^3^/g]	micropore surface area [m^2^/g]	external surface area [m^2^/g]	BET [m^2^/g]	average pore diameter [nm]
TiOSO_4_	0.00	0.00	0.92	0.92	10.52
TS_2	0.00	0.00	7	6.97	17.30
TS_1	0.11	232	117	349	2.60
MA	0.23	478	25	502	2.10
NaT_1	0.18	402	28	430	2.10
NaT_2	0.00	0.00	5	5	18.3

The synthesis described uses titanyl sulfate dihydrate as the basic raw material, and the entire synthesis is carried out in an aqueous environment. Titanyl sulfate is a commercially available byproduct of the production of titanium white pigment by the sulfate process; the other raw materials are common commercially available environmentally acceptable chemicals. No hazardous waste is generated during the synthesis, e.g., the resulting sodium sulfate solution is completely harmless and industrially recoverable if large quantities are produced, as confirmed by the toxicity assessment described in ref (32).

The material described in this publication exhibits properties suitable for the synthesis of complex compounds in which various inorganic and organic substances such as various anions (e.g., SO_4_^2–^, halide anions, ClO^4–^, Fe(CN)_6_^4–^, and others), amines, organic acids, mono-, bi-, and trivalent cations, and a variety of other substances are bound to the metatitanitic acid skeleton. The complexes prepared in this manner may be used, for example, for the sorption of other difficult to sorb cations or anions.

### 2.2 Methods of Characterization

The following methods have been used for morphological, structural, and chemical characterization of the product: scanning electron microscopy (SEM/EDS), transmission electron microscopy (TEM), surface area (BET), X-ray diffraction (XRD), Raman spectroscopy, thermal analysis coupled with mass spectrometry (TA-MS), and zeta potential measurements. Details including experimental conditions are described in the Supporting Information Characterization Methods.

## 3 Results and Discussion

### 3.1 Materials Description

**Figure 1:**
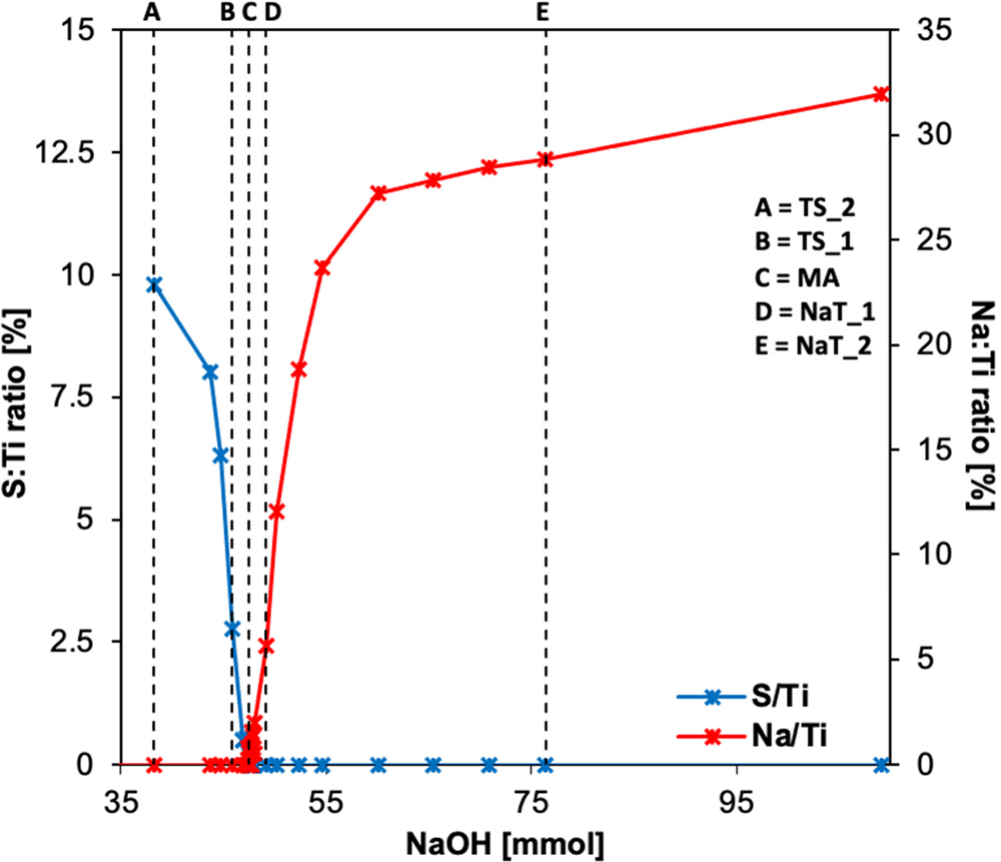
Dependence of the volume of sodium hydroxide added (in mmol) on Na (wt %) or S (wt %), respectively.

The preparation method used in this work is characterized by the use of the titanyl sulfate as a template for the determination of the size/shape of the prepared titania aggregates. The crystals of titanyl sulfate dihydrate in the base aqueous solution (in our case sodium hydroxide) form, under specific reaction conditions, a product which can be described as a hydrated titanium dioxide with varying sodium or sulfate contents depending on the ratio of entering reactants. Figure 1 shows the dependence of Na:Ti and S:Ti ratios on the addition of NaOH.

During the reaction, the loosely bound SO_4_^2–^ anions of titanyl sulfate are replaced by OH^–^ from the solution while the particle character remains unchanged. Depending on the pH, the excess charge is balanced by Na^+^ cations. The reaction can be described as a pseudomorphic transformation, as the shape and dimensions of the micrometer-size particles of the starting material are perfectly preserved. The mechanism can be described as follows:
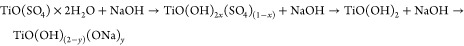


In our experience, *x* can reach values of 0.9–1 (at higher values the products are soluble), and the maximum value of *y* can reach 0.3. The experiment is conducted by placing solid dihydrate of titanyl sulfate in aqueous NaOH solution at ∼0 °C. At values of *x* < 0.9, dissolution of titanyl sulfate occurs. At *x* = 1, metatitanic acid is formed, and at *x* > 1, sodium titanates are formed with varying sodium contents up to *y* ≈ 0.3, depending on the amount of NaOH added.

For further analysis, five samples were selected from different areas of the graph, representing pure metatitanic acid (MA, indicated in Figure 1 as sample C), sulfur containing products (TS_1 and TS_2, indicated in Figure 1 as samples A and B), and sodium containing products (NaT_1 and NaT_2, indicated in Figure 1 as samples D and E).

As our experiments show, the maximum amount of sodium that can be incorporated into the microrods is achieved with the addition of approximately 60 mmol of NaOH into the reaction mixture formed from 24.5 mmol of titanyl sulfate dihydrate. The resulting molar Na:Ti ratio is approximately 0.35, and the product does not change significantly with higher additions of NaOH (Table 1).

Lower amounts of added NaOH reduce the amount of sodium in the final product until it completely disappears around 47.5 mmol. A point can be found where the sample has all of the S ions washed out and no sodium is yet present. The point where neither sodium nor sulfur is present describes the formation of hydrated titanium dioxide or metatitanic acid. A decrease in the added NaOH further results in the presence of sulfur in the product. If less than 35 mmol of NaOH is added to the reaction mixture, then dissolution processes of the particles take place. These processes are out of the scope of this article.

Although the titanyl sulfate dihydrate is soluble in water within tens of minutes, the residues after the reaction with aqueous sodium hydroxide are not soluble in water and are mechanically highly resistant, even to high-energy ultrasound. Such materials eliminate the substantial problem of materials based on nanostructured hydrated titanium dioxide (difficult-to-filter), as the prepared materials have no tendency to form colloids.

### 3.2 Materials Characterization

#### 3.2.1 Surface Area and Porosity Measurements

**Figure 2:**
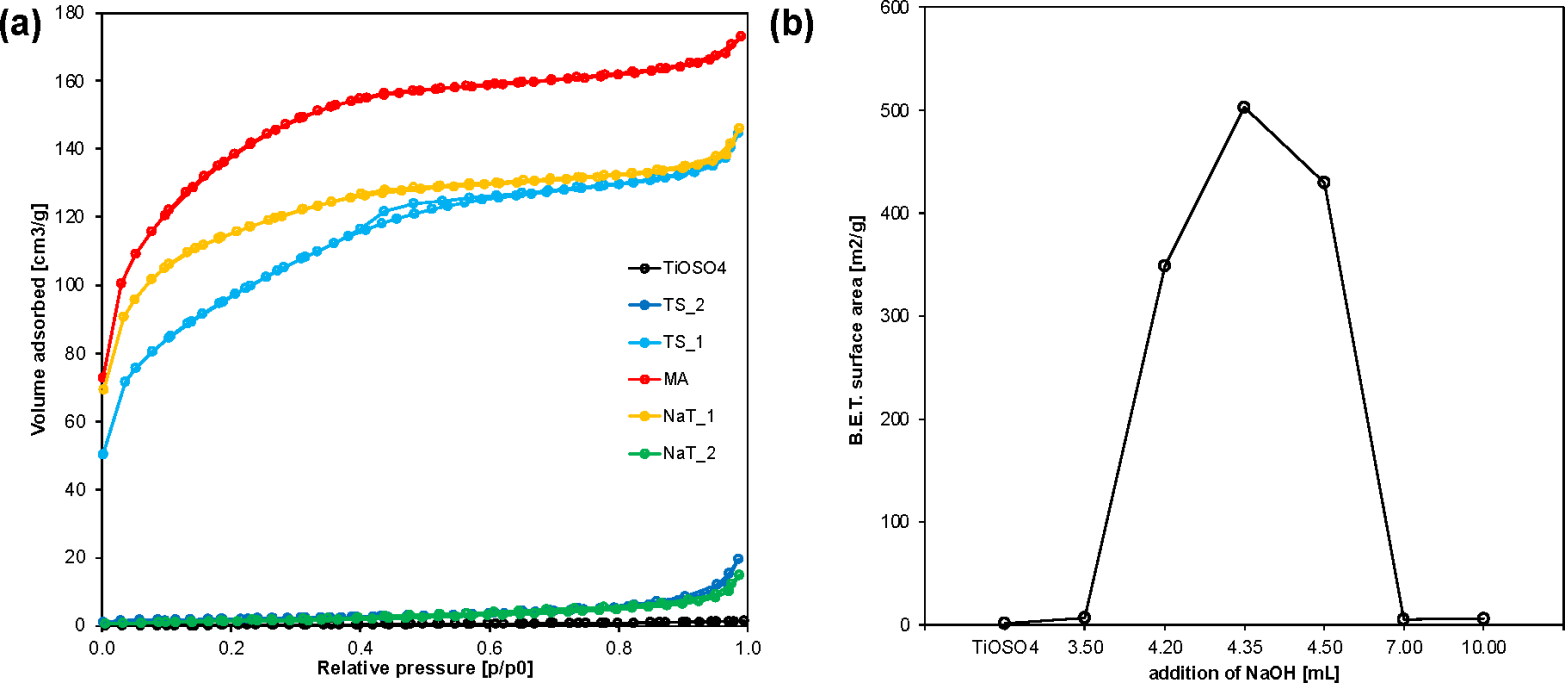
Isotherms of low-temperature (77 K) nitrogen adsorption and desorption on selected samples (a) and graphical representation of the BET surface area (b).

The surface area and porosity of the selected samples were studied by nitrogen adsorption/desorption, and the resulting isotherms are shown in Figure 2a. Some features can be observed directly from these isotherms. The TS_2 and NaT_2 samples show type II isotherms (as classified by IUPAC^34^), which are typical for macroporous or nonporous materials. The samples TS_1, MA, and NaT_1 show type I(b) and II combined isotherms, which are typical for microporous materials with mesopores present as well. The nitrogen uptake at high relative pressures, which is observed in all of the analyzed samples, corresponds to nitrogen condensation in macropores or interparticle voids.

The surface area of the samples as calculated using the BET method is presented in Table 1 and in Figure 2. There is a clear trend in the surface area measured in the studied samples, with maximum surface area values in samples with no or very low Na or S content. It has been suggested^35^ that complete Na replacement by protons causes structural collapse in titanate materials, thus resulting in the decrease of surface area. This, however, was not observed in our study. As can be seen in Section 3.2.5, the microrod morphology is very well preserved for all the Na:Ti ratios, including the lamellar structure.

According to Morgado et al.,^36^ the specific surface area in these materials can be caused either by morphological changes (such as collapse of the structure) or by decrease of skeletal density due to changes in chemical composition (as the BET calculation depends on the weight of the sample). Further analysis is needed to fully understand the reasons for such behavior.

The BET method was applied to express the surface size of mesoporous materials, the t-plot method was chosen to express the surface size of microporous materials, and the results are presented in Table 1.

**Figure 3:**
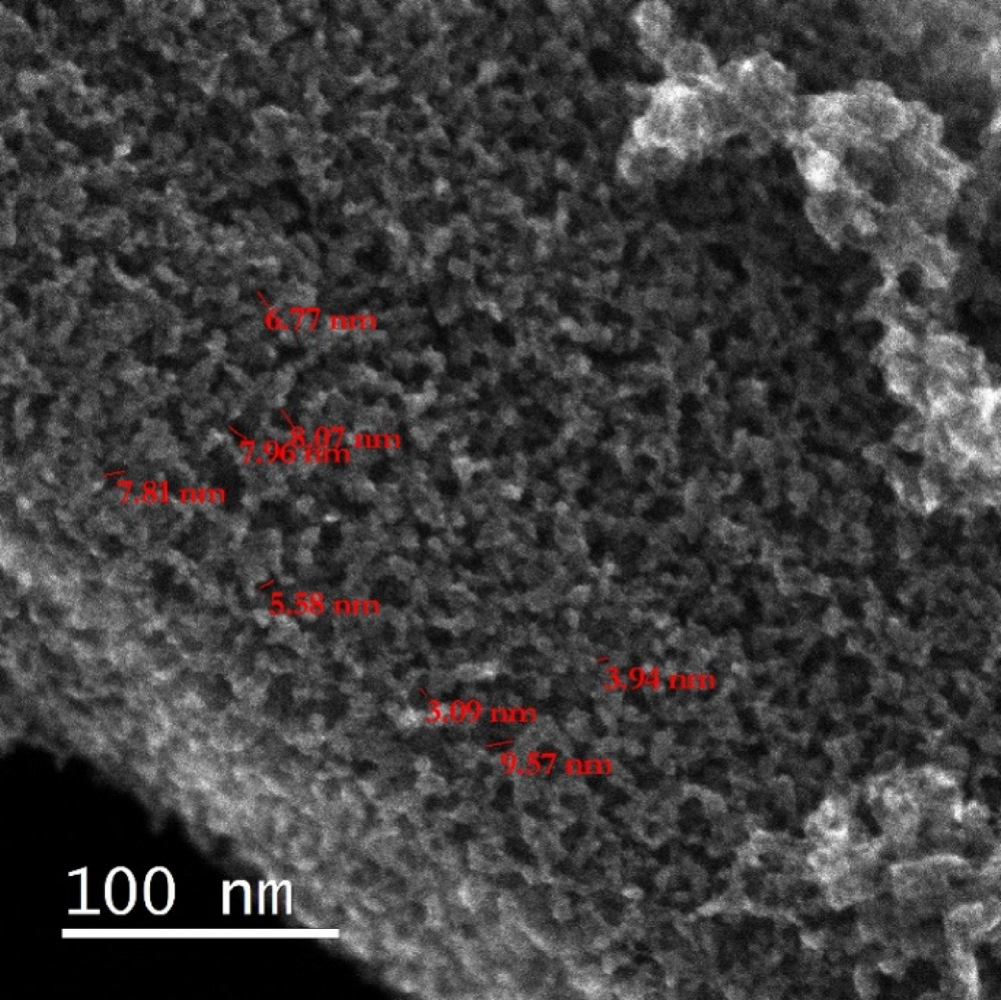
SEI-STEM image of the MA.

The micropores of pure metatitanic acid (sample MA) can also be observed in the STEM micrograph (Figure 3). A structure is clearly visible, showing nanoparticles with dimensions in units of nanometers surrounding pores, most of which are ≤5 nm in diameter. This structure can also be seen in more detail in Section 3.2.6, where it is also discussed further. Since the material is very imperfectly crystalline with only hints of anatase crystallinity forming, the high reactivity of the material and its consequent utility in various syntheses can be expected.

#### 3.2.2 Zeta Potential

**Figure 4:**
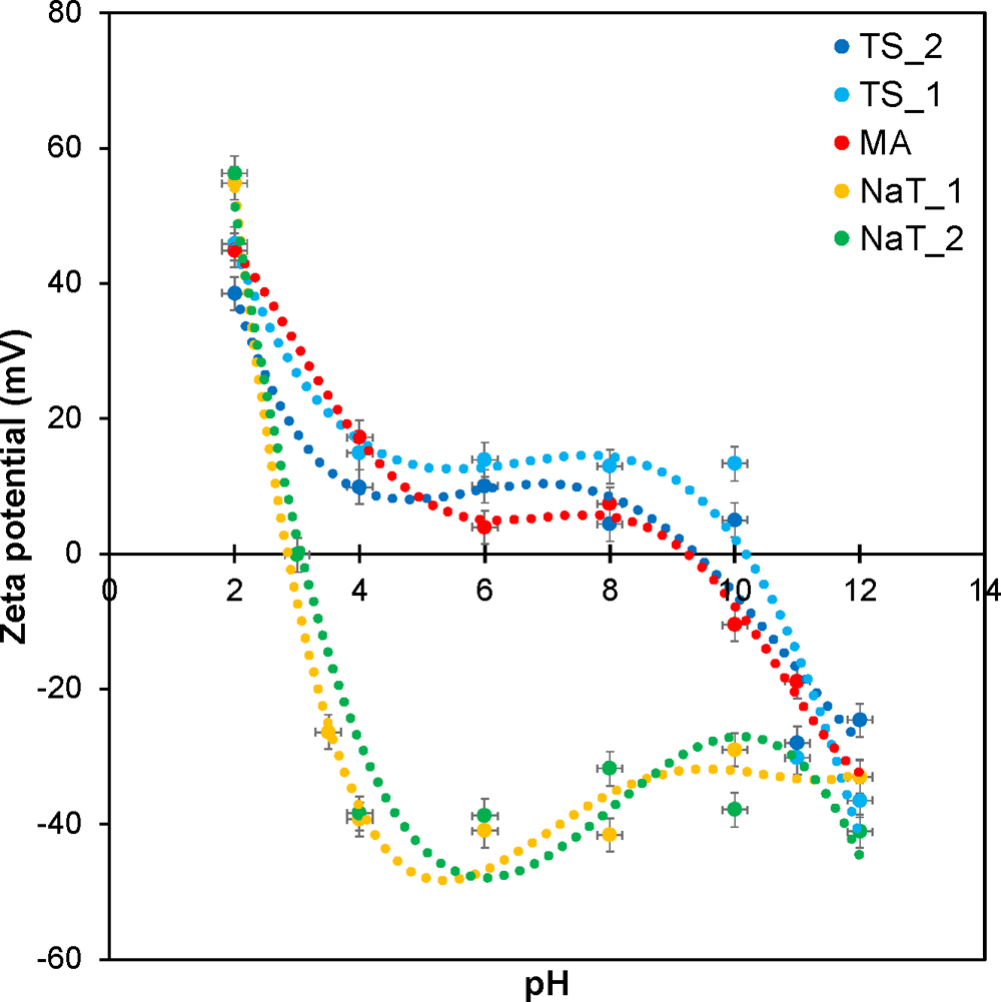
Dependence of zeta potential on pH for samples TS_2 (red), TS_1 (gray), MA (yellow), NaT_1 (blue), and NaT_2 (green).

The dependence of the zeta potential values on pH has a classic S curve for metatitanic acid (MA) (Figure 4). The values of the zeta potential lie in the positive region up to pH values around 8, and the isoelectric point is likely around pH 9. This relatively wide range of pH values in which the zeta potential is positive is likely due to the highest content of titanium cations (Table 1). Titanium cations can compensate for the addition of hydroxide and OH^–^ ions, respectively. With further addition of hydroxide, the zeta potential values are already in negative numbers.

Samples containing sulfur in the structure (TS_1 and TS_2) do not differ much in terms of zeta potential values from the MA sample. A more significant difference can be observed for sample TS_1, whose zeta potential values have shifted to the positive region compared to those of the MA sample. The isoelectric point of sample TS_1 is around pH 10. The titanium content is slightly lower, but sample TS_1 is characterized by the highest external surface (Table 1) and thus has the highest counterion availability for charge compensation. The samples with sodium contents designated as NaT_1 and NaT_2 have a completely different zeta potential course. The isoelectric points of both samples lie around pH 3. The content of titanium ions is lower compared to the MA sample, and above all, titanium is more tightly bound into the crystallographic structure. The samples contain phases of anatase, whose surface is extremely negatively charged and can thus compensate for the increasing content of H^+^ ions after the addition of acid.

#### 3.2.3 Thermal Decomposition

**Figure 5:**
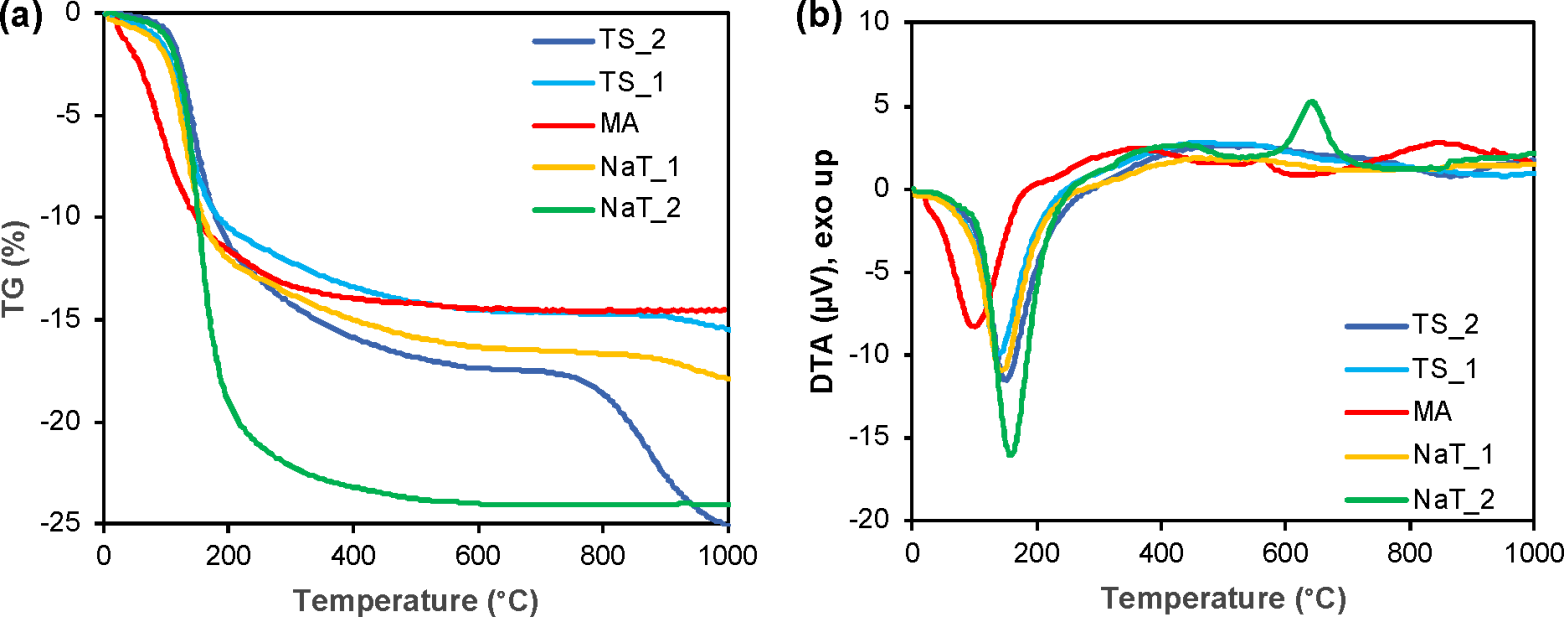
Thermal decompositions of prepared samples, TG curves (a), and DTA curves (b).

The thermal transformations of prepared TiO_2_ precursors with rod-shaped structure were followed by simultaneous TG/DTA coupled with evolved gas detection until 1000 °C, as can be seen in Figure 5. The mass losses for all samples are in in the range of 15–25%. The sulfates are evolving as expected for samples TS_1 and TS_2 (confirmed by *m*/*z* = 64 evolving at 880 °C), and samples with higher addition of NaOH are free of residual sulfates, proving that the washing procedure was sufficient. Exothermic reactions of samples MA, NaT_1, and NaT_2 take place above 400 °C, and according to high-temperature HT-XRD (summarized data in Table S1), the MA sample crystallizes into anatase at 400 °C and then rutile is present in the structure at 1000 °C. Sodium containing samples are transformed into mixtures of sodium titanates and anatase.

#### 3.2.4 XRD and Raman Spectroscopy

**Figure 6:**
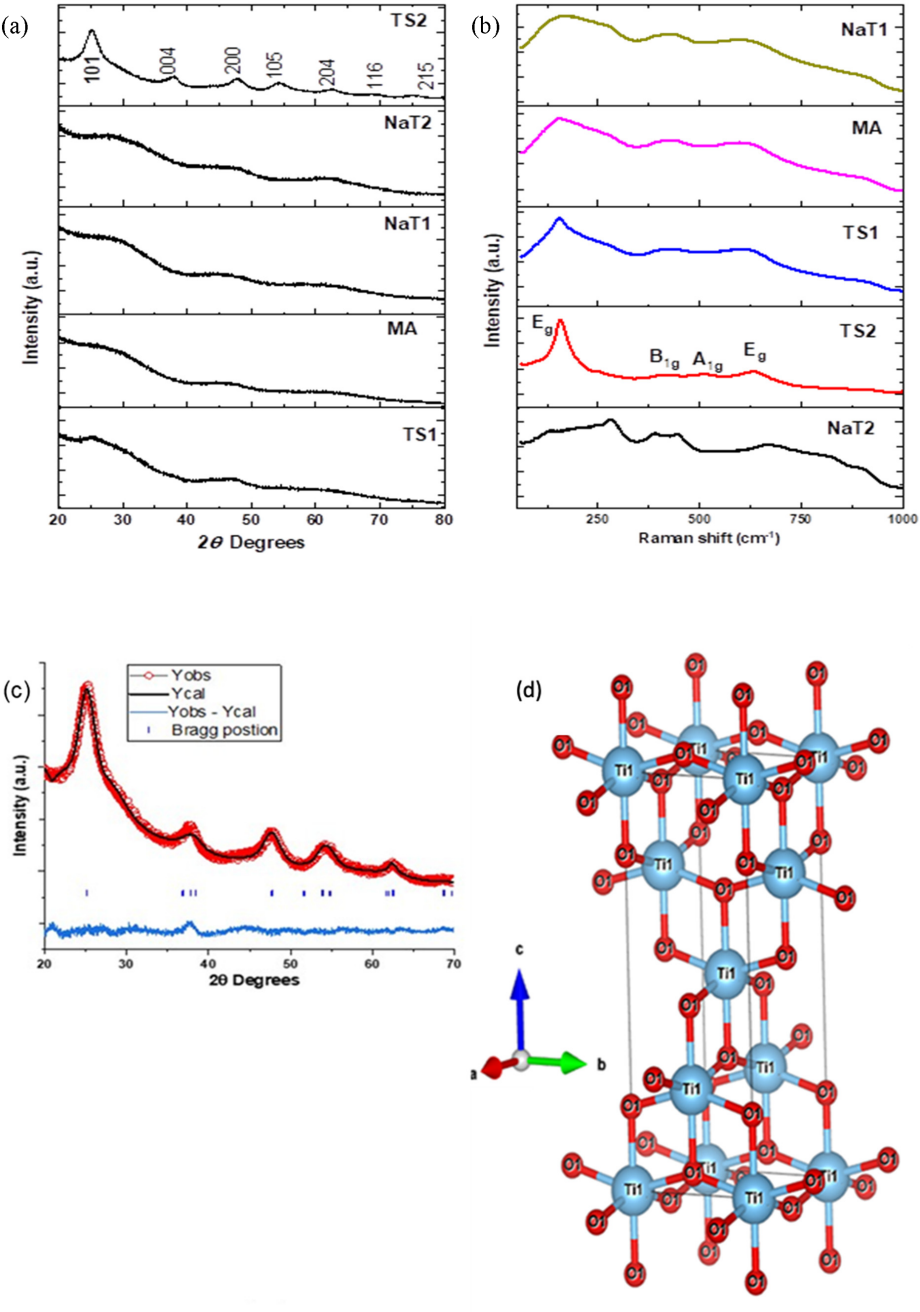
Powder XRD (a) and Raman spectra (b) of the prepared samples. (c) The fit of the Rietveldt refinement and (d) the anatase-type structure of metatitanic acid.

The X-ray diffraction (XRD) patterns for all of the samples are shown in Figure 6a. The sample TS_2 shows the successful formation of pure-phase tetragonal anatase (JCPDS 89-4921). All other samples also have the same phase with broad peaks due to the nanostructure nature of these samples.^37^ Also, the high-resolution TEM images confirm the interference fringes, suggesting the presence of small crystallites. Diffraction patterns of all of the studied samples are present in Figure S1. Although the diffraction rings are rather diffuse, the radial integral intensities of these patterns revealed crystalline phases. Additionally, Figure 6b provides structural insights through Raman spectroscopy. Four distinct Raman-active modes of anatase TiO_2_ with symmetries of Eg, B1g, A1g, and Eg were identified at 155, 410, 512, and 630 cm^–1^, respectively. These characteristic vibrational frequencies and intensity ratios confirm the pure anatase TiO_2_ phase. The results correspond to our spectra, suggesting the presence of titanate H_*x*_Ti_2–*x*/4*x*_/_4_O_4_·H_2_O of lepidocrocite-type layered structure.^38^ The slight shift of several bands together with high complexity in the region of 120–300 cm^–1^ may be due to the presence of sodium ions.

The Rietveld FullProf method is used to further investigate the exact crystal structure using crystal symmetry I41/amd (space group no. 141) in a tetragonal structure, as shown in Section 3.2.5. The lattice parameters are found to be *a* = *b* = 3.8174 Å; *c* = 9.4711 Å; and *V* = 138.0185 Å^3^. The fitting parameters *R*_p_ and *R*_wp_ are 3.89 and 4.96, respectively. The Raman spectra for the TS_2 sample are shown in Figure 6d and again confirm the anatase phase for the sample. Figure 6d depicts the crystal structure of metatitanic acid, as obtained by Vesta software.

#### 3.2.5 SEM

**Figure 7:**
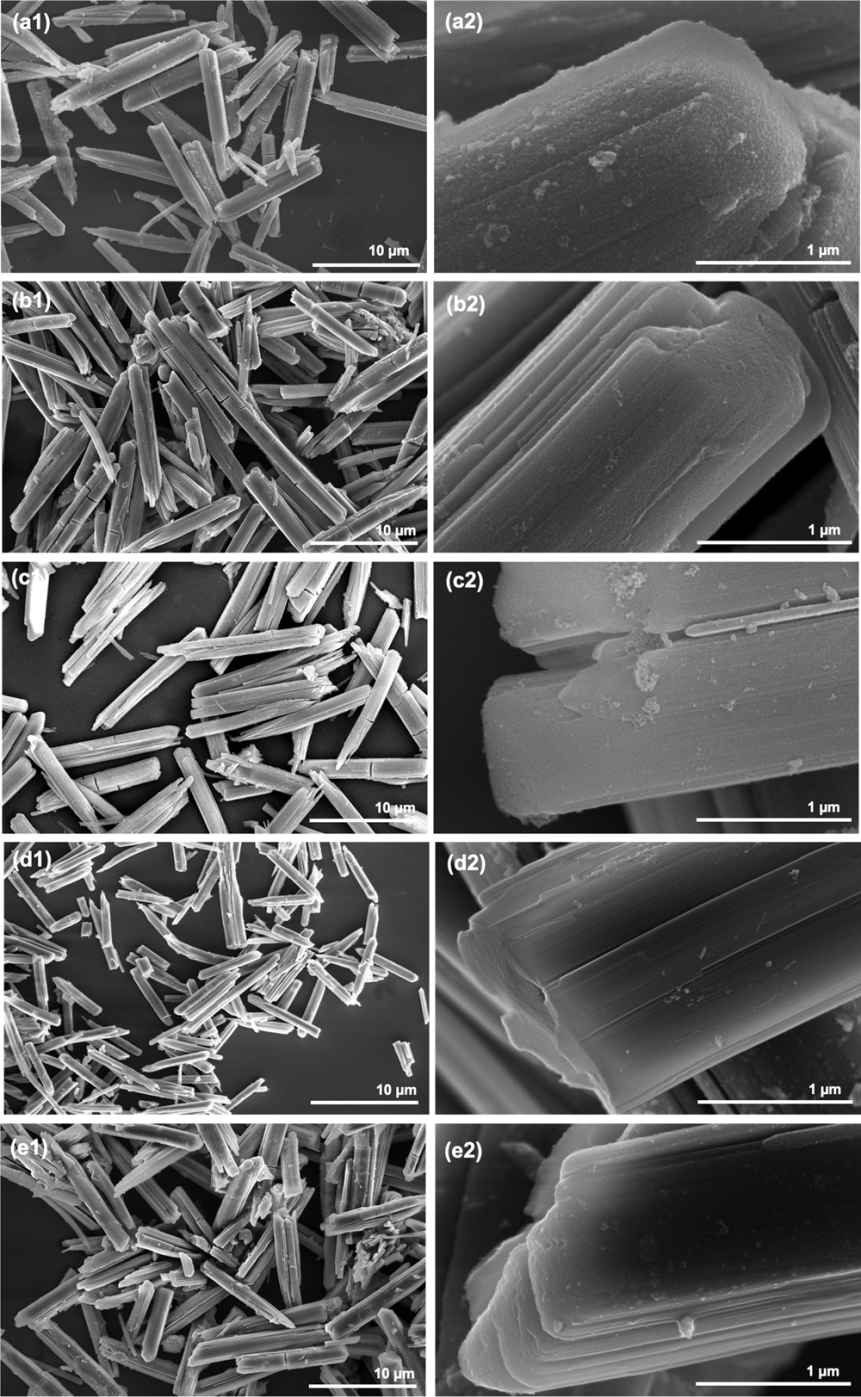
SEM micrographs of TS_2 (a1 and a2), TS_1 (b1 and b2), MA (c1 and c2), NaT_1 (d1 and d2), and NaT_2 (e1 and e2) at different magnifications.

**Figure 8:**
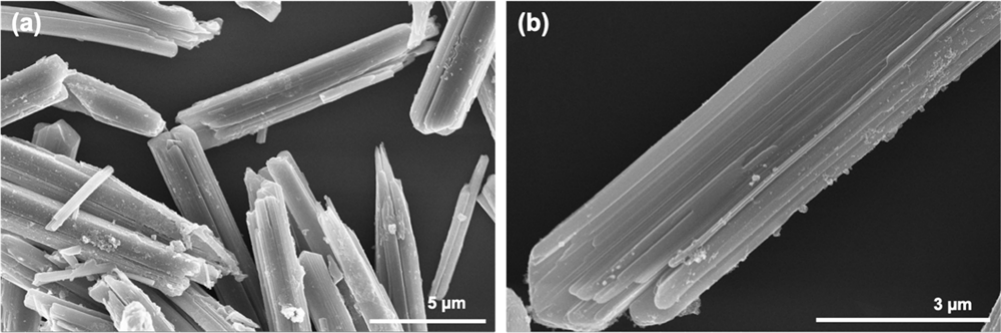
SEM micrographs of starting titanyl sulfate dihydrate at different magnifications: (a) 20000x and (b) 65000x.

The scanning electron microscopy measurements revealed that the particles of all prepared samples are of regular rods with the size of 10–15 μm × 2 μm (Figure 7) and have preserved the size and morphology of the starting titanyl sulfate dihydrate including such details like layered structure (Figure 8), as previously described by ref (27). The EDS analysis data are summarized in Table 2 as an average value of five measurements.

**Table 2:** Elemental Composition of Titanyl Sulfate Dihydrate and of the Samples Formed from 24.5 mmol of Titanyl Sulfate Dihydrate, in at. %, Normalized Results

	sample description		elemental composition			
	mL	mmol	Na	S	Ti	O
TS_2	3.50	38.2	0	2.89	23.90	73.21
TS_1	4.20	45.9	0	0.81	29.42	69.77
MA	4.35	47.5	0	0	27.74	72.26
NaT_1	4.50	49.1	1.58	0	28.55	69.86
NaT_2	7.00	76.4	7.74	0	26.93	65.32
TiOSO_4_·2H_2_O	-	-	-	13.35	14.51	72.14

#### 3.2.6 TEM Measurements of MA

**Figure 9:**
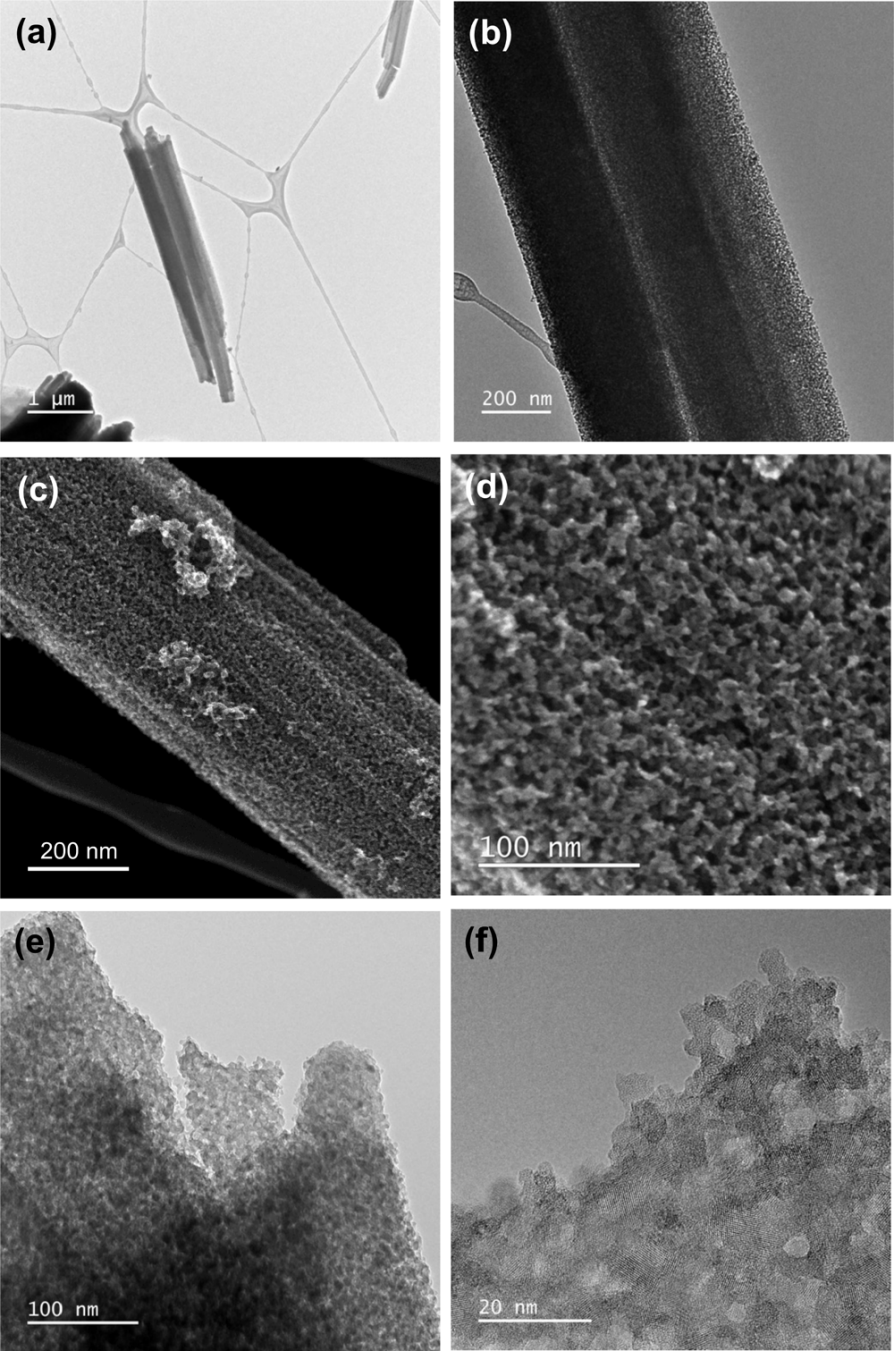
Low-magnification TEM images of rods of MA (a–f) and SEI-STEM detailed images of rods (c and d).

**Figure 10:**
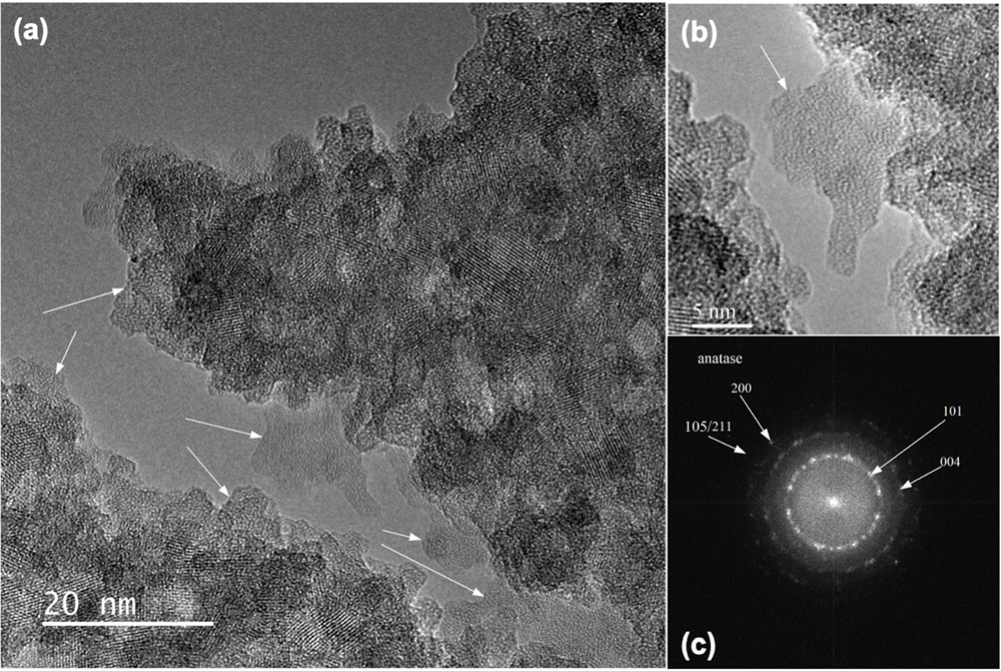
HRTEM detail of rods, illustrating the partially crystalline character of rods with randomly oriented crystallites exhibiting sizes from 4.5 to 10 nm. Arrows indicate the amorphous phase (a) and detail of the amorphous phase (b). (c) FFT pattern acquired from (a).

Low-magnification TEM images are presented in Figure 9. They show that the powder sample consists of rod-like objects with diameters from 100 nm up to 2.5 μm and lengths of several micrometers (most of them were up to 5 μm). As can be observed from the SEI-STEM and TEM images in Figures 9c–9f, the rods are polycrystalline and exhibit a random orientation of crystallites, which manifests the FFT ring pattern in Figure 10c gained from Figure 10a.

The size of crystallites ranges from 4.5 to 10 nm (Figure 10a). The relevant FFT pattern in Figure 10c acquired from Figure 10a shows that the crystallites consisted of an anatase TiO_2_ polymorph. The determined interplane distances of 0.350, 0.238, 0.190, and 0.169 nm obtained by measurements of the most intense rings in the FFT pattern can be assigned to the 101, 004, 200, and 105/211 reflections of the anatase TiO_2_ polymorph according to PDF 21-1272.

**Figure 11:**
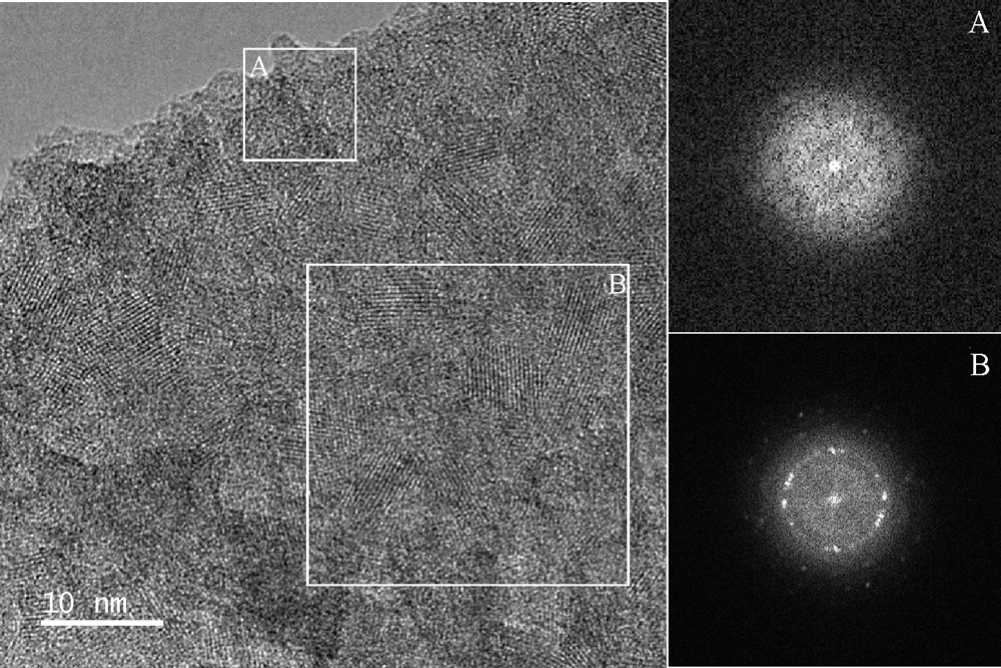
(a) Layer of the amorphous phase at the surface of the nanorod. FFT taken from square “A” demonstrates the presence of the amorphous phase. (b) The diffuse halo ring and SAED circle (taken from square “B”) manifest the coexistence of amorphous and crystalline phases.

Except from anatase nanocrystals, the amorphous phase was revealed in the sample, as indicated by arrows in Figure 10a. A detailed image of the amorphous object squared by “B” is in Figure 11b.

An amorphous phase was found to cover the surfaces of nanorods, as shown in Figure 11a. A relevant FFT image consisting of a diffusion halo confirms the presence of the amorphous phase in area “A” in Figure 11b. The diffuse halo and SAED circle acquired from area “B” indicate the coexistence of the amorphous and crystalline anatase phases inside the nanorods, which can be seen in Figure 11c.

**Figure 12:**
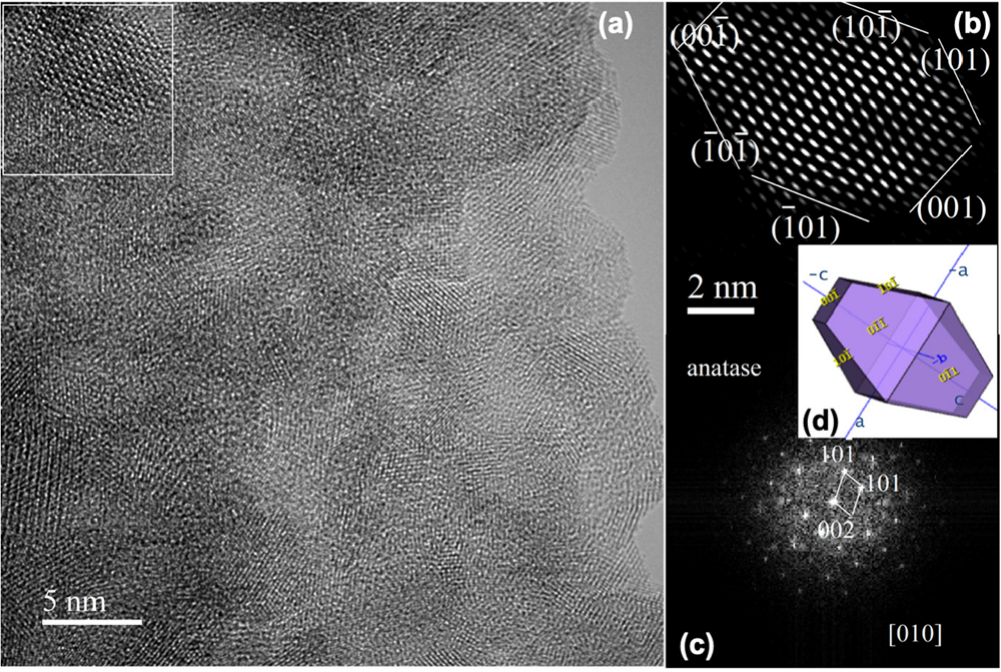
HRTEM image of the near-surface structure of the rod (a), IFFT image of the anatase single crystal oriented along the [010] direction (b), FFT pattern of a selected anatase nanocrystal with highlighted reflections (c), and schematic image of the 3D morphology of the anatase nanocrystal (d).

Some of the anatase nanocrystals are well faceted, as highlighted in the square in Figure 11a. An example of a 2D projection of a well faceted anatase nanocrystal having a size around 8.6 nm and characteristic truncated dipyramidal morphology is apparent in this image. Inverse FFT imaging (IFFT) in Figure 12b provides the anatase nanocrystal in more detail. A scheme of the 3D morphology of the anatase nanocrystal is given in Figure 12d. By evaluation of the relevant FFT pattern, shown in Figure 12c, it was determined that the crystal is oriented by the [010] direction along the primary electron beam and is predominantly faceted by {101} and {001} type planes.^39^ The measured angle 43.3° between two marked 101 and 101 type reflections is in accordance with the theoretical angle 43.4° between the (101) and (101) planes of anatase. Moreover, the measured angle of 111.8° agrees with 111.7° between the (101) and (001) planes of anatase.

#### 3.2.7 TEM Measurements

TEM investigations of other samples reveal that the morphology of the particles of titanyl sulfate (sample TSD) is closely preserved in the resulting products, and the TEM images are presented in Figure S2. The high-magnification image showed the porous character of the products. Very broad diffraction rings show the presence of crystallization nuclei in all samples.

**Figure 13:**
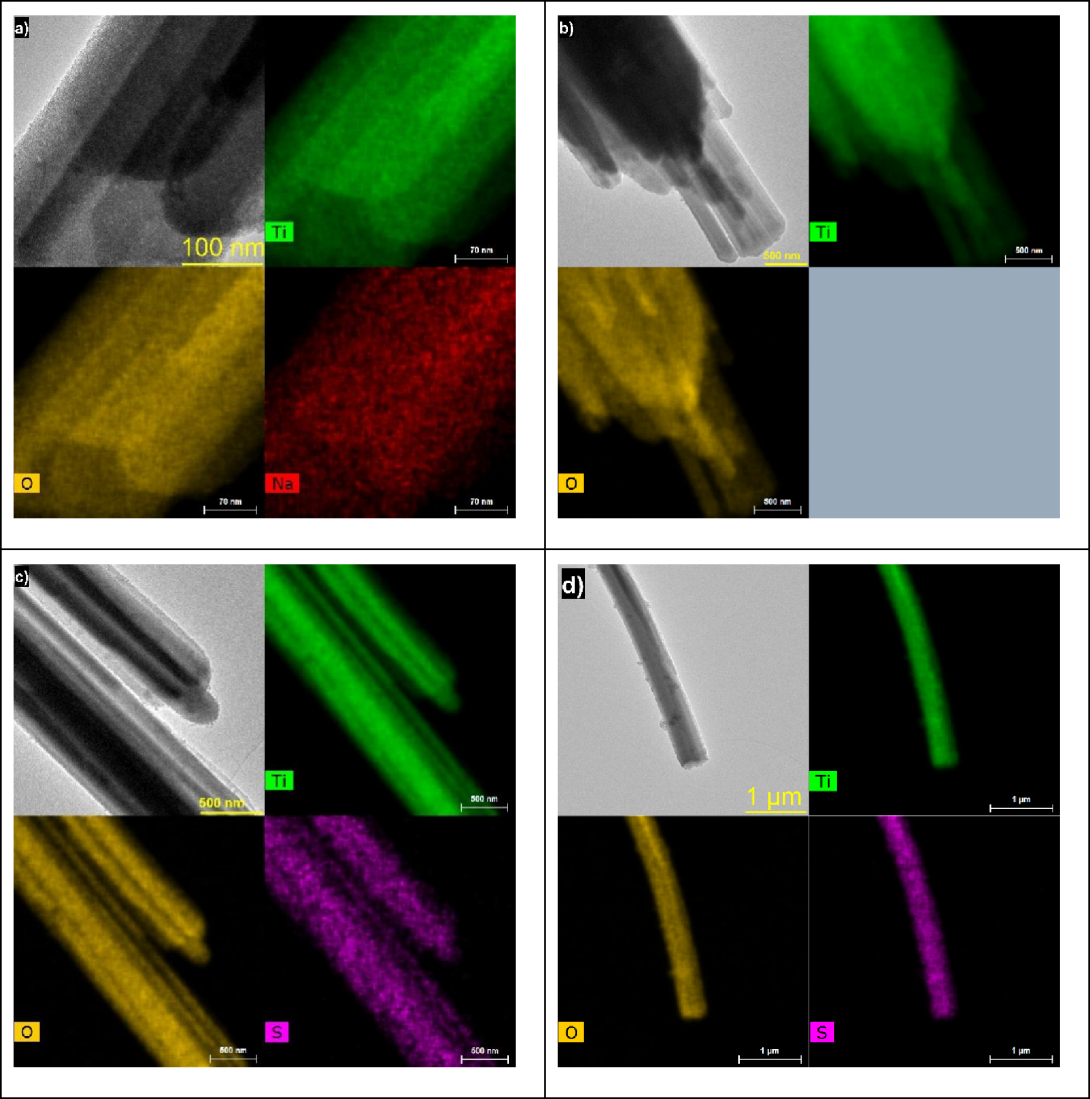
TEM/EDS mapping of (a) NaT_1, (b) MA, (c) TS_1, and (d) TS_2.

Mapping of the prepared samples (Figure 13) showed the homogeneous composition of all rods, all in good agreement with observations by SEM/EDS measurements above.

## 4 Conclusions

The described process produces pseudocrystals containing either sulfate anions or alkali cations. Depending on the ratio of the alkali metal to the titanyl sulfate, substances with different contents of alkali cations or sulfate anions are formed, and with certain ratios, pure hydrated titanium dioxide is obtained, according to the analysis corresponding to the formula TiO(OH)_2_ (metatitanic acid). The products are generally amorphous, with indications of crystallinity corresponding to the anatase structure. The material is highly porous, with the highest surface area values of ∼500 m^2^·g^–1^ being reached in the metatitanic acid sample. The detailed structure of the prepared metatitanic acid was characterized by HRTEM, and it was found that in addition to the anatase nanocrystals, the sample also contains a substantial share of amorphous phase. The prepared samples are mechanically highly resistant, even by high-energy ultrasound, and have no tendency to form colloids.

## Supporting Information Available

Characterization methods description, diffraction patterns of all prepared samples, HT-XRD analysis results, and TEM images of all prepared samples (PDF)
