# Supplementary material for: A Metatitanic Acid Particulate Xerogel: Green Synthesis, Structure Determination, and Detailed Characterization
Source: Inorg Chem. 2024 Jun 29;63(28):12730–9. doi: 10.1021/acs.inorgchem.4c00369 (PMC11256744; doi:10.1021/acs.inorgchem.4c00369)
Supplement: Supplementary file 1 — ic4c00369_si_001.pdf [file ic4c00369_si_001.pdf]

Supporting information

A metatitanic acid particulate xerogel: green synthesis, structure determination and detailed characterization

Monika Motlochová[1,2], *Xenia Vislocká*[1], *Sven Lidin*[2], *Mária Čaplovičová*[3], *Roman Maršálek*[4], *Jan Šubrt*[1]

[1]Institute of Inorganic Chemistry of the Czech Academy of Sciences, 250 68 Řež, Czech Republic

[2]Centre for Analysis and Synthesis, Lunds Universitet, Naturvetarvägen 14, Lund 222-61, Sweden

[3]Slovak University of Technology in Bratislava, Faculty of Material Science and Technology, Centre for Nanodiagnostics of Materials, Vazovova 5, Bratislava 81243, Slovakia

[4]Department of Chemistry, Faculty of Science, University of Ostrava, CZ-701 03 Ostrava, Czech Republic

*Corresponding author:
Tel. +420 311 236 907, E-mail: motlochova@iic.cas.cz (Monika Motlochova)

Characterization methods

The following methods were used for morphological, structural, and chemical characterization of the product: scanning electron microscopy (SEM/EDS), transmission electron microscopy (TEM), surface area measurements (BET), thermal analysis (TG/DTA) and zeta potential measurements.

Morphology of the powdered samples was studied using scanning electron microscope JEOL JSM 6510LV (high vacuum mode, accelerating voltage 25 kV), and elemental analysis were carried out with an Oxford Instruments Energy Dispersive X-ray (EDX) detector. EDX analyses were acquired and evaluated in the INCA software package.

Detailed phase analysis including imaging and electron diffraction were carried out on a transmission electron microscope (TEM) JEOL JEM 3010 operated at 300 kV (LaB6, cathode, point resolution 1.7 Å). Investigation was also performed using a double-corrected TEM JEOL JEM ARM 200cF , which was operated at an accelerating voltages of 80 kV and 200 kV (STEM HAADF spatial resolution: 0.078 nm at 200 kV). Energy Dispersive X-ray Spectroscopy (EDX) was performed using a large angle JEOL JED-2300 T CENTURIO SDD (silicon drift) detector, boasting a solid angle of up to 0.98 sr and a detection area of 100 mm^2. Images were recorded on a Gatan CCD camera with resolution of 1024 × 1024 pixels and 2048x2048 pixels using the Digital Micrograph software package. The powder samples were dispersed in ethanol and the suspension was treated in ultrasound for 2 min. A drop of very dilute suspension was placed on a holey-carbon coated Cu-grid and allowed to dry by evaporation at ambient temperature.

Thermogravimetry (TG), differential thermal analysis (DTA) and evolved gas analysis (MS) were carried out in apparatus SetSys Evolution (SETARAM). Thermoanalytical measurements were carried out in an inert atmosphere (argon, 60 cm3 min-1), in an open crucible made of a-Al2O3, and initial sample mass was approximately 20 mg (crushed to fine powders). Samples were heated from the room temperature to 1000 °C with heating rate of 10 °C min-1 which enabled a detailed study of the whole decomposition process.

Surface area was determined by the BET method using a Quantachrome Nova 4200e instrument. Nitrogen adsorption was car- ried out at −196 °C. Before analysis, the non-annealed samples were pretreated at RT under vacuum for 35 h.

XRD measurements were carried out using a PANalytical X´Pert PRO diffractometer equipped with a conventional X-ray tube (CuKα 40 kV, 30 mA, line focus) in transmission mode. An elliptic focusing mirror, a divergence slit 0.5 °, an anti-scatter slit 0.5 ° and a Soller slit of 0.02 rad were used in the primary beam. A fast linear position sensitive detector PIXcel with an anti-scatter shield and a Soller slit of 0.02 rad were used in the diffracted beam. All patterns were collected in the range of 18 to 88 deg. 2theta with the step of 0.013 deg and 400 sec/step producing a scan of about 2.5 hours. Qualitative analysis was performed with HighScorePlus software package (PANalytical, The Netherlands, version 3.0e), DiffracPlus software package (Bruker AXS, Germany, version 8.0) and JCPDS PDF-4 database. For quantitative phase

analysis DiffracPlus Topas (Bruker AXS, Germany, version 4.2) with structural models based on ICSD database was used. This program permits to estimate the weight fractions of crystalline phases by means of Rietveld refinement procedure. The estimation of the size of crystallites was performed on the basis of Scherrer formula as implemented within the DiffracPlus Topas software.

Raman spectra were recorded using a dispersive Raman Nicolet Almega XR instrument (excitation wavelength 473 nm, output 10 mW, spectral range 100-1300 cm-1, resolution 6,5-13 cm-1). Software package Omnic 8.2.0.387 (Thermo Fishes Scientific, inc.) was used for analysis of the measured data. The Raman spectra of anatase and rutile shown for comparison were taken from Nicodom spectra library.

The zeta potential of suspensions was measured using a Zetasizer Nano ZS (Malvern Instruments Ltd., GB). Suspensions intended for measurement were prepared by mixing 10 mg of solid sample and 10 ml of distilled water. The pH of the suspension was adjusted by adding hydrochloric acid or sodium hydroxide solution. Before the measurement, the samples were sonicated for 5 min. Subsequently, 1 ml of the suspension was inserted into the measuring cell. Zeta potential was calculated from electrophoretic mobility using the Smoluchowski equation. All measurements were performed three times.

Results

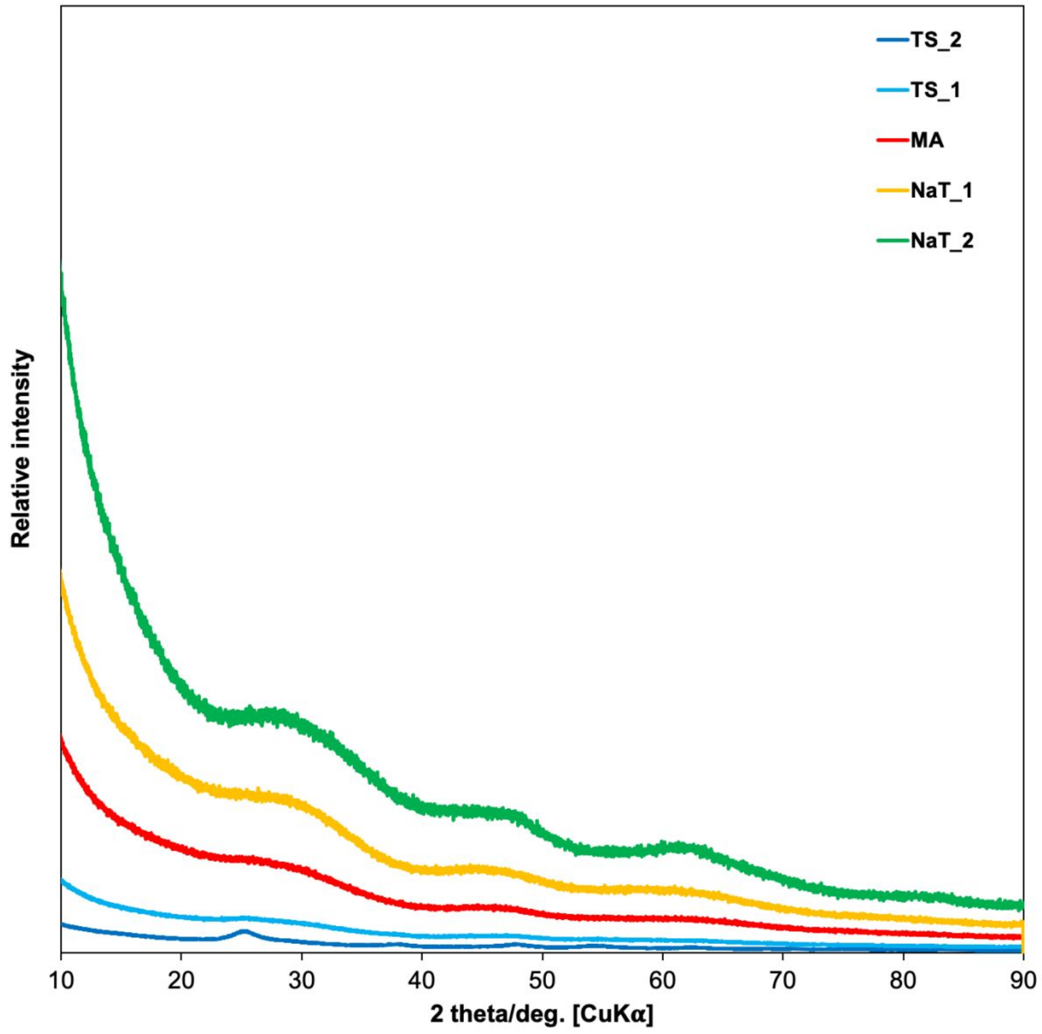

Figure S1 Diffraction patterns of all prepared samples.

Table S1 HT-XRD analysis of prepared samples.

Sample	Temp. of first crystals / [°C]		
	Anatase	Rutile	Titanate
TS_2	450	850	×
TS_1	350	800	×
MA	350	850	×
NaT_1	350	700	750
NaT_2	×	×	550

NaT_2

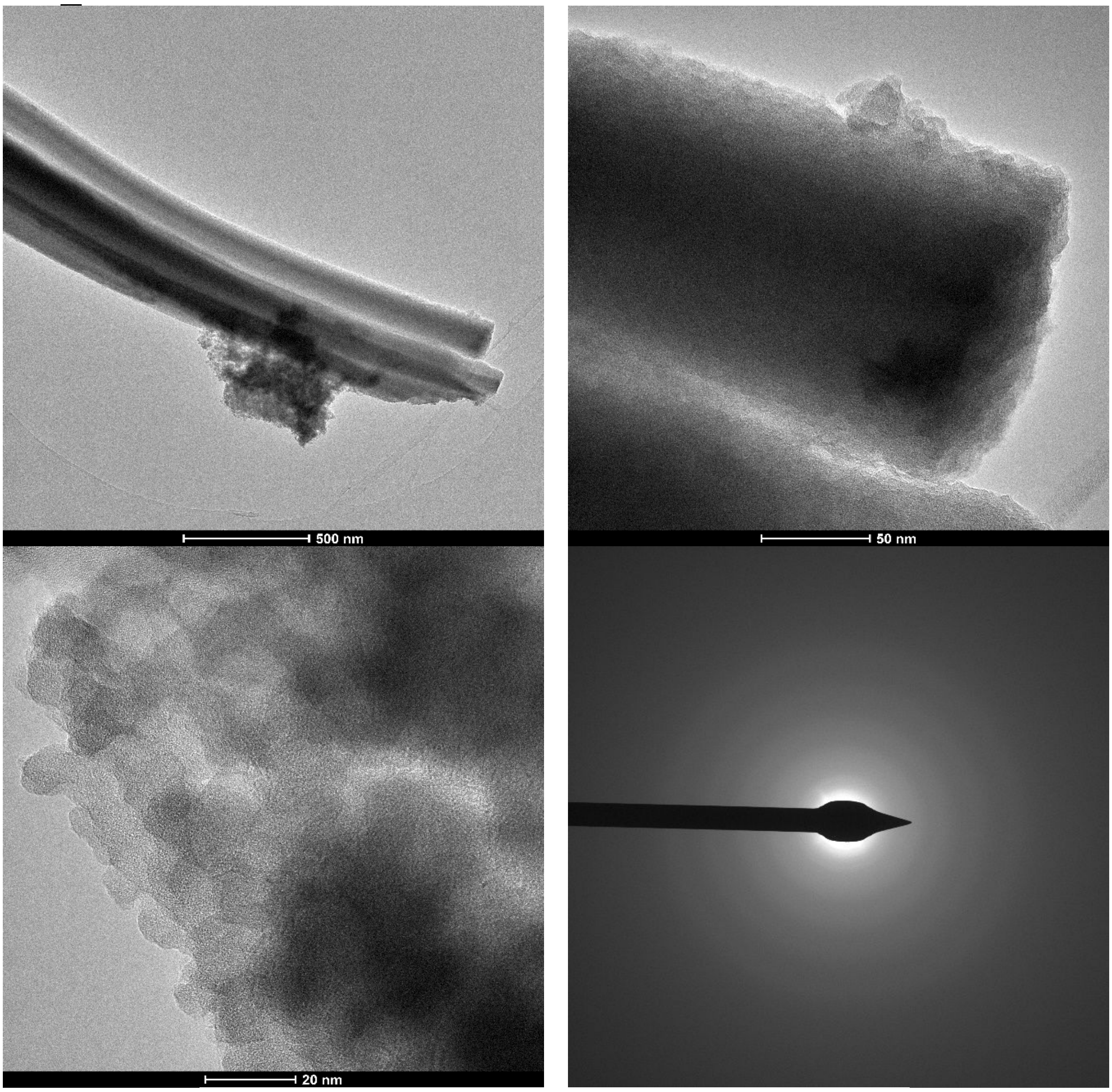
500 nm
50 nm
20 nm

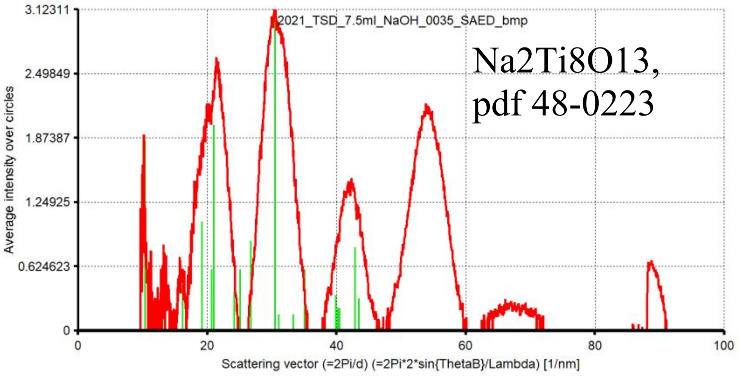
2021_TSD_7.5ml_NaOH_0035_SAED_bmp
Na2Ti8O13,
pdf 48-0223
3.12311
2.49849
1.87387
1.24925
0.624623
0
Average intensity over circles
0
20
40
60
80
100
Scattering vector (=2Pi/d) (=2Pi*2*sin{ThetaB}/Lambda) [1/nm]

NaT_1

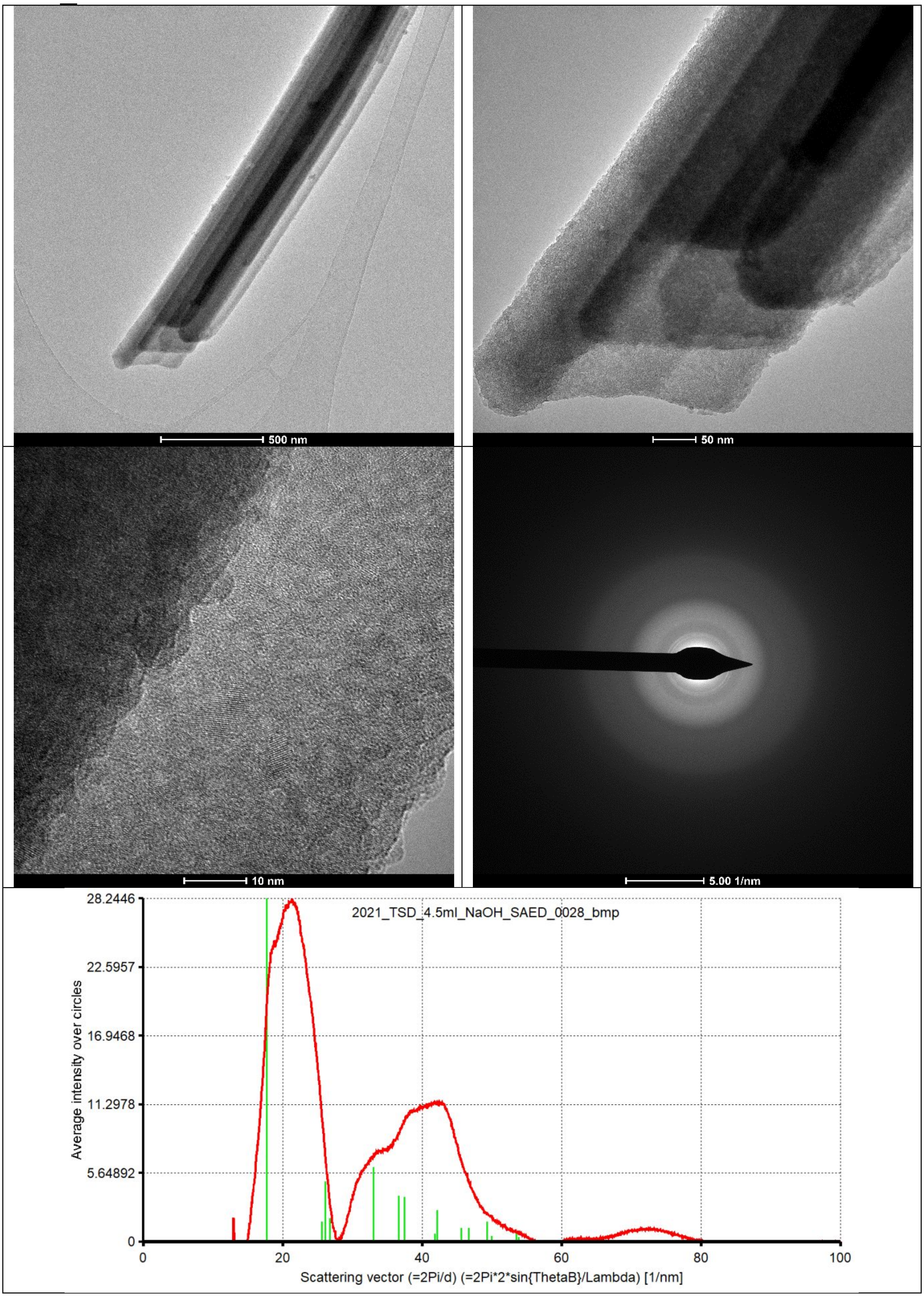
500 nm
50 nm
10 nm
5.00 1/nm
2021_TSD_4.5ml_NaOH_SAED_0028_bmp
Average intensity over circles
28.2446
22.5957
16.9468
11.2978
5.64892
0
0
20
40
60
80
100
Scattering vector (=2Pi/d) (=2Pi*2*sin{ThetaB}/Lambda) [1/nm]

MA

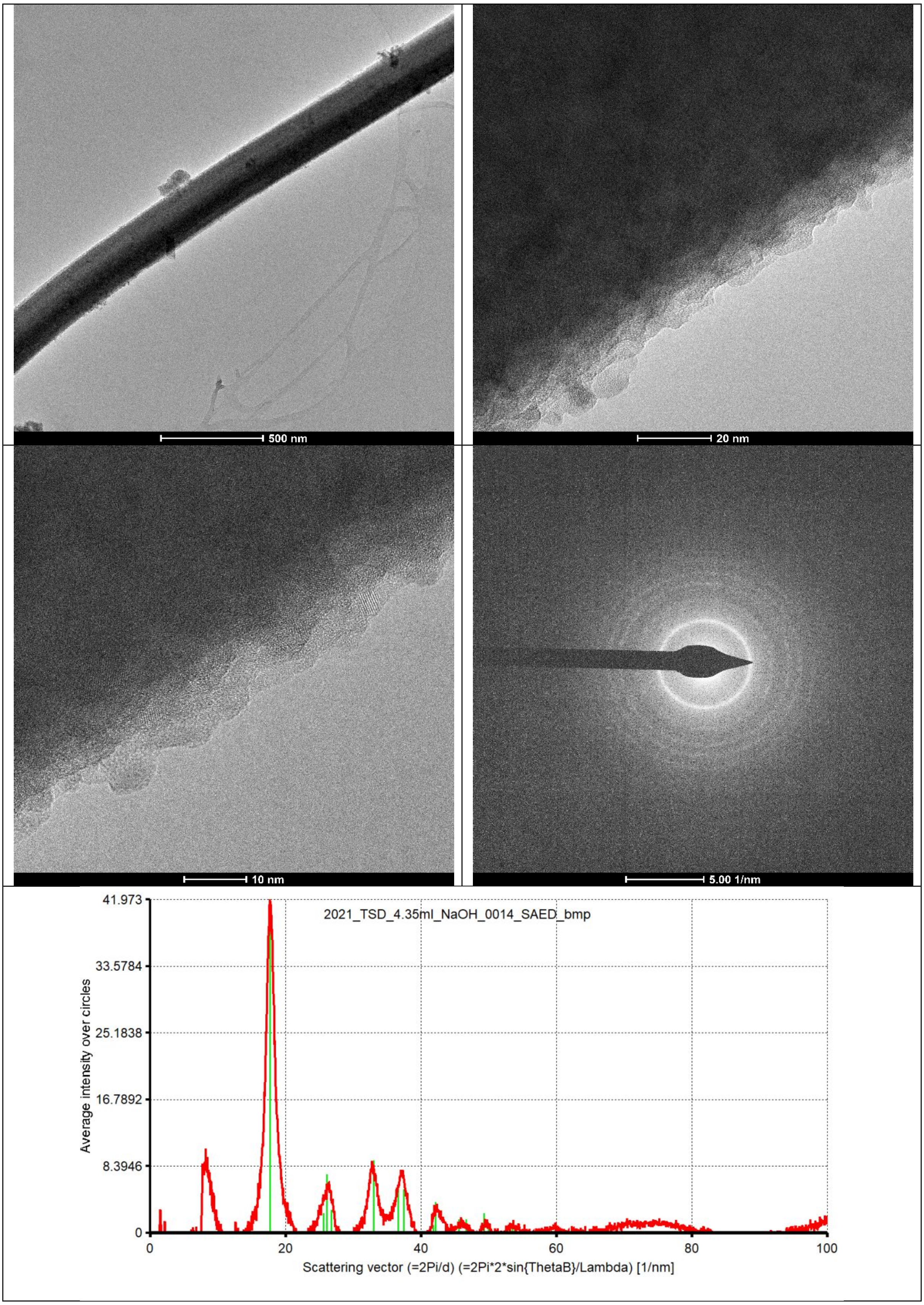
500 nm
20 nm
10 nm
5.00 1/nm
2021_TSD_4.35ml_NaOH_0014_SAED_bmp
Average intensity over circles
41.973
33.5784
25.1838
16.7892
8.3946
0
0
20
40
60
80
100
Scattering vector (=2Pi/d) (=2Pi*2*sin{ThetaB}/Lambda) [1/nm]

TS_1

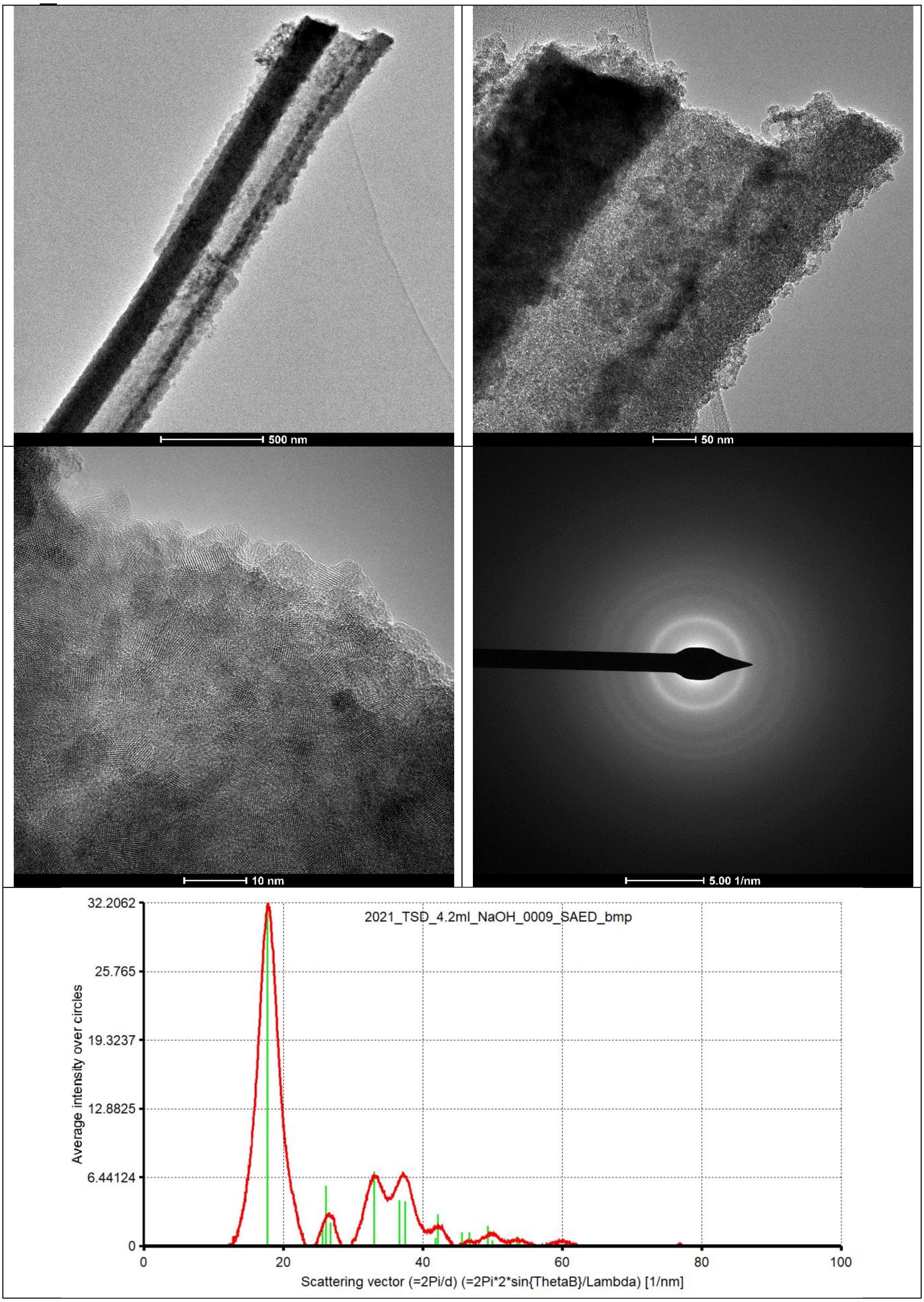

500 nm
50 nm
10 nm
5.00 1/nm
2021_TSD_4.2ml_NaOH_0009_SAED_bmp
Average intensity over circles
32.2062
25.765
19.3237
12.8825
6.44124
0
0
20
40
60
80
100
Scattering vector (=2Pi/d) (=2Pi*2*sin{ThetaB}/Lambda) [1/nm]

TS_2

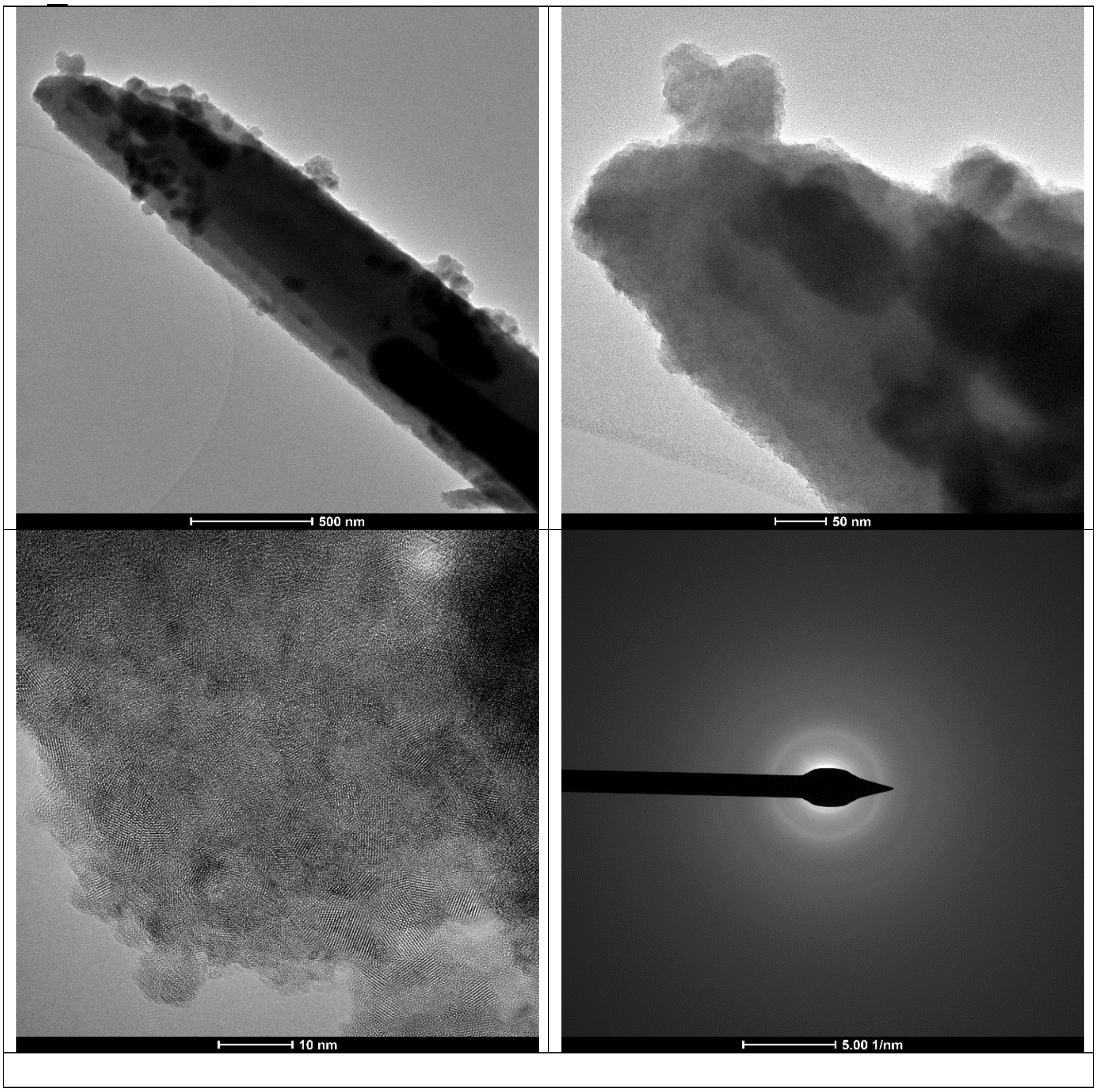

Figure S2 TEM images of all prepared samples.
